# Correlation of diabetic retinopathy with oxidative biomarkers among diabetic patients

**DOI:** 10.6026/973206300211301

**Published:** 2025-06-30

**Authors:** Anunitha R., Rashmi G.

**Affiliations:** 1 Department of Ophthalmology, Sri Devaraj Urs Medical College, Tamaka, Kolar, Karnataka, India

**Keywords:** Microvascular complications, antioxidant effects of bilirubin, oxidative stress, Type 2 diabetes mellitus

## Abstract

The correlation between serum uric acid, bilirubin levels and diabetic retinopathy (DR) in 68 patients with diabetes were studied. Higher grades of DR were significantly associated with elevated fasting blood sugar, postprandial blood sugar, HbA1c and serum uric acid levels, while serum bilirubin levels demonstrated an inverse relationship. A majority of patients had diabetes duration of more than one year, emphasizing the role of chronic hyper-glycemia in DR progression. Monitoring serum uric acid and bilirubin may aid in early detection and assessment of DR severity. However, these biomarkers may have limited utility in predicting the initial development of DR.

## Background:

Diabetes mellitus (DM) is a chronic metabolic disorder that disrupts glucose regulation, increasing the risk of systemic complications [1]. In type 2 diabetes mellitus (T2DM), metabolic disturbances contribute to microvascular and macrovascular complications [2]. Hyperglycemia drives biochemical alterations, including lipid metabolism disturbances and oxidative stress, which are central to diabetic retinopathy (DR), a leading cause of vision impairment accounting for approximately 5% of global blindness cases [3, 4]. Known DR risk factors include prolonged diabetes duration, poor glycemic control hypertension and hyperlipidaemia [5]. Bilirubin's vascular protective effects, first proposed by Najib-Farah in 1937, are attributed to its potent antioxidant and anti-inflammatory properties [6, 7]. Conversely, elevated serum uric acid (SUA) levels, resulting from the evolutionary loss of uricase activity, are associated with hyperinsulinemia and insulin resistance [8]. Given the role of oxidative stress in diabetic retinopathy, it is essential to explore the contribution of high serum uric acid (SUA) to microvascular complications in T2DM, as it promotes oxidative stress via NLRP3 inflammasome activation [9]. Therefore, it is of interest to describe the relationship between serum bilirubin and uric acid levels with diabetic retinopathy, highlighting their potential role as oxidative stress markers for early detection and risk evaluation.

## Materials and Methods:

A cross-sectional study was conducted at the Department of Ophthalmology from August 2023 to August 2024, following Central Ethics Committee approval (No. SDUMC/KLR/IEC/579/2023-24 dated 02/02/2024). Inclusion criteria were patients aged over 18 years with T2DM. Exclusion criteria included patients with hypertension, anemia, leukemia, hypercoagulable states, prior kidney diseases, severe infections, malignancy, or prior radiation therapy. After informed consent, all patients underwent a complete ophthalmological examination. Parameters recorded included visual acuity, slit-lamp biomicroscopy, indirect ophthalmoscopy and fundus examination. Blood samples were collected for fasting blood sugar (FBS), postprandial blood sugar (PPBS), HbA1c, serum uric acid and total bilirubin. The biological reference ranges were: FBS 74-106 mg/dL, PPBS 80-140 mg/dL, HbA1c <6.5% (good control), 6.5-7% (fair), 7-9% (suboptimal), >9% (poor), serum uric acid 3.5-8.5 mg/dL and serum bilirubin 0.2-1.3 mg/dL. Diabetic retinopathy was graded using the Early Treatment Diabetic Retinopathy Study (ETDRS) classification. Data were analyzed using SPSS version 22. Statistical significance was set at p<0.05.

## Results and Discussion:

The study enrolled 68 patients with a mean age of 62.5 ± 10.5 years. Males comprised 52.9% and females 47.1%, minimizing gender bias. Fundus findings included mild non-proliferative diabetic retinopathy (NPDR) (35.2%), moderate NPDR (22.3%), severe NPDR (7.3%) and normal fundus (35.2%). Most patients (54.8%) had diabetes duration less than 10 years, while 25.8% had diabetes for over 10 years. Glycemic indices were elevated: mean FBS 154.5 ± 64.02 mg/dL, PPBS 201.5 ± 89.1 mg/dL and HbA1c 8.8 ± 2.06%. The mean serum uric acid (SUA) level was 5.4 ± 1.80 mg/dL and mean serum bilirubin was 0.59 ± 0.43 mg/dL. Table 1 (see PDF) summarizes the comparison of blood investigation levels and fundus examination findings, showing significant associations between glycemic indices, SUA and bilirubin with DR severity (ANOVA test, p<0.05) [Serum Uric Acid and Bilirubin as Biomarkers for Diabetic Retinopathy Severity, Department of Ophthalmology, 2023-2024]. Higher FBS (196.1 ± 91.5 mg/dL), PPBS (201.2 ± 91.3 mg/dL) and HbA1c (9.9 ± 3.2%) levels were significantly associated with severe NPDR (p<0.05). Serum uric acid levels were highest in severe NPDR (7.4 ± 1.4 mg/dL, p=0.0474), while serum bilirubin levels were lowest compared to normal fundus (0.7 ± 0.5 mg/dL, p=0.0001). Guo *et al.* reported that patients with proliferative diabetic retinopathy (PDR) had significantly higher SUA levels than those without DR (p<0.001), suggesting SUA as a marker of oxidative stress [1].

Liu *et al.* found that diabetes duration >10 years increased DR risk (OR: 2.14, 95% CI: 1.45-3.16, p<0.001), consistent with our finding of severe NPDR in 25.8% of patients with prolonged diabetes [2]. Hu *et al.* demonstrated that higher SUA levels were linked to vision-threatening DR (OR: 1.20, 95% CI: 1.05-1.38, p=0.01), with stronger associations in males [5, 14]. Ren *et al.* reported a synergistic effect of high SUA and low TBIL on microvascular complications (OR: 3.417, 95% CI: 1.635-7.139), aligning with our observation of elevated SUA (7.4 ± 1.4 mg/dL) and reduced bilirubin (0.63 ± 0.4 mg/dL) in severe NPDR [4]. Bilirubin's protective role is evident. Cho *et al.* observed an inverse relationship between serum bilirubin and DR risk (p<0.05), with lower levels linked to increased severity [3]. Zhu *et al.*'s meta-analysis confirmed a negative nonlinear relationship between bilirubin levels and DR progression (HR: 0.70, 95% CI: 0.54-0.89) [6]. Karuppannasamy *et al.* reported that higher bilirubin levels were associated with lower DR incidence (OR: 0.55, 95% CI: 0.31-0.98) [7]. Prabhavathi *et al.* found that lower bilirubin levels correlated with increased oxidative stress markers in DR (p<0.01), supporting our findings of reduced bilirubin in severe NPDR (0.63 ± 0.4 mg/dL) [17]. Ding *et al.* highlighted bilirubin's role in mitigating retinal oxidative damage and vascular leakage [15]. The interplay between SUA and bilirubin is critical. Kang and Yang noted that elevated SUA promotes oxidative stress via NLRP3 inflammasome activation and cytokine production (*e.g.*, TNF-α, IL-6), exacerbating retinal ischemia [9]. Haydinger *et al.* suggested that bilirubin's antioxidant properties, including reactive oxygen species scavenging, counteract these pathways [10]. López-Contreras *et al.* reported elevated oxidative stress markers like 8-hydroxy-2'-deoxyguanosine in DR, with bilirubin offering protection [11]. Wu *et al.* noted that mitochondrial dysfunction, driven by oxidative stress, contributes to DR, with SUA exacerbating retinal damage [12]. Diabetes duration significantly influences DR severity. Zhang *et al.* found that diabetes duration >10 years increased severe DR risk (p<0.001), driven by hyperglycemia-induced AGEs and endothelial dysfunction [8]. Our study supports this, with 25.8% of patients with >10 years diabetes duration showing severe NPDR, higher SUA and lower bilirubin. Cecilia *et al.* suggested that biomarkers such as SUA, combined with clinical assessments, may improve DR risk evaluation [13]. Vinuthinee-Naidu *et al.* found SUA levels correlated with retinal nerve fiber layer thinning (p=0.002), indicating neurodegeneration [16]. Gao *et al.* proposed that antioxidant therapies targeting oxidative stress could mitigate DR progression, supporting bilirubin's protective role [18]. Kundu *et al.* further substantiated the role of oxidative stress in the pathogenesis of diabetic retinopathy by demonstrating significantly elevated oxidative stress markers, including malondialdehyde (MDA), in DR patients compared to controls [19]. Our study indicates that SUA and bilirubin are valuable for assessing DR severity but have limited utility for predicting DR onset, consistent with Zhu *et al.*'s findings [6]. Monitoring these biomarkers could enhance risk stratification and guide interventions to prevent DR progression, particularly in patients with prolonged diabetes duration.

## Conclusion:

Serum bilirubin and uric acid levels may serve as biomarkers for assessing the severity of diabetic retinopathy (DR), rather than predicting its onset. Elevated uric acid and reduced bilirubin levels were associated with greater disease severity, underscoring the role of oxidative stress in DR pathogenesis. Incorporating these markers into routine monitoring could aid in early detection, risk stratification and prevention of DR progression and related complications.
